# Talaromycosis from Wuhan: two-case report and literature review

**DOI:** 10.3389/fcimb.2024.1347677

**Published:** 2024-03-11

**Authors:** Zhiyuan Yao, Zhou Pan, Guang Li, Zhaomin Liao, Zhen Yu, Liying Zhan, Wenfang Xia

**Affiliations:** Department of Critical Care Medicine, Renmin Hospital of Wuhan University, Wuhan, Hubei, China

**Keywords:** acquired immune deficiency syndrome, renal transplantation, *Talaromyces marneffei*, Talaromycosis, glucose-6-phosphate dehydrogenase deficiency

## Abstract

**Background:**

Talaromycosis is a serious opportunistic infectious disease caused by *Talaromyces marneffei*, which mostly occurs in immunocompromised patients. The disease is mainly prevalent in tropical countries and regions of Southeast Asia and South Asia, but non-endemic areas also have patients with Talaromycosis. The disease has no characteristic clinical manifestations and is difficult to diagnose. Delayed diagnosis often leads to death.

**Case presentation:**

Both patients had cellular immunodeficiency. Case 1 had a history of acquired immune deficiency syndrome, and case 2 had a history of renal transplantation and glucose-6-phosphate dehydrogenase deficiency. They all had fever, anemia, fatigue, and skin lesions. Case 1 had gastrointestinal bleeding, enlarged lymph nodes, and hepatosplenomegaly. Case 2 had cough and dyspnea. Both patients had thrombocytopenia and hypoalbuminemia; an increased neutrophil ratio, procalcitonin, and C-reactive protein; and abnormal liver function and coagulation dysfunction. Case 1 sputum culture, blood culture, and bronchoalveolar lavage fluid were positive for *T. marneffei*. *T. marneffei* was detected in the blood culture of case 2, with infection of *Candida parapsilosis* and *Pneumocystis jirovecii*. Chest computed tomography scan mainly showed pulmonary exudative lesions. Although these two patients were actively treated, they died of poor efficacy.

**Conclusion:**

Talaromycosis has an insidious onset, long course, atypical clinical symptoms, imaging performance and laboratory results, difficult diagnosis, and high mortality. Therefore, it is important to promptly consider and treat Talaromycosis in immunocompromised patients upon infection in order to reduce mortality.

## Background

Talaromycosis (TSM) is an invasive deep fungal disease caused by *Talaromyces marneffei*. *T. marneffei* was previously classified as *Penicillium* but has since been reclassified in *Talaromyces* with advances in taxonomy and related techniques (Tsang et al., 2018, 2019). In October 2022, the World Health Organization (WHO) released the “Fungal Priority Pathogens List,” which ranked *T. marneffei* as a medium priority (WHO fungal priority pathogens list to guide research, development and public health action, n.d.). *T. marneffei* mainly exists in tropical countries in South and Southeast Asia (Cao et al., 2019). In China, 99.4% of TSM cases were reported in southern China (Hu et al., 2013), especially in Guangdong and Guangxi provinces (26.5% and 12.5%, respectively) (Qin et al., 2020). Due to the increase in population mobility, the incidence of TSM in non-endemic areas has gradually increased (Cao et al., 2019). For example, the incidence in Hubei Province is about 1.2%–2.3% (Qin et al., 2020), but the incidence is much lower than that in the southern region, and most patients have a history of sojourn in endemic areas. *T. marneffei* is a mold structure at 25°C and a yeast structure at 37°C (DiSalvo et al., 1973). It is the only fungus in the *Talaromyces* that is thermally dimorphic and the only member of the genus that has been confirmed to cause invasive infections in humans and animals (Tsang et al., 2018). The bamboo rat is the only non-human natural host of *T. marneffei* (Hu et al., 2013). However, there is no evidence that the fungus can be directly transmitted from bamboo rats to humans, and there is no human-to-human transmission (Limper et al., 2017). It is generally stated that that the infection is caused by contact with the conidial spore in the soil through the digestive tract or respiratory tract (Wang et al., 2018), and the incidence is related to humidity (Tsang et al., 2018). TSM likely to occur in patients with acquired immune deficiency syndrome (AIDS), and *T. marneffei* infection is one of the three representative infections in patients with AIDS (Yuen et al., 1994). The mortality of adult patients with AIDS infected with *T. marneffei* is 12%~21% (WHO fungal priority pathogens list to guide research, development and public health action, n.d). The prevalence of *T. marneffei* infection in human immunodeficiency virus (HIV)–positive patients in China has also gradually increased in recent years. From 2011 to 2017, the prevalence of *T. marneffei* infection increased from 15.7% to 18.8% in Guangdong Province (Ying et al., 2020), 16.1% in Guangxi Province (Jiang et al., 2019), and 4.8% in Wuhan (Zheng et al., 2015), Hubei Province. In recent years, there have been more reports of *T. marneffei* infection in non-AIDS patients. Most of these patients have underlying diseases that lead to immunodeficiency, such as organ transplantation, interferon-γ autoantibodies, drug abuse, lymphoma, and leukemia (Lee et al., 2012, 2014; Chan et al., 2015; Tse et al., 2015; Guo et al., 2020; Yang et al., 2021b; Gupta et al., 2022; Lu et al., 2020), but *T. marneffei* also occurs in non-immunodeficiency patients (Chen et al., 2020). Non-HIV–infected patients are often misdiagnosed as tuberculosis (80.7%) or bacterial pneumonia (20.5%) after *T. marneffei* infection (Guo et al., 2019). They are also misdiagnosed as intestinal tuberculosis (Yang et al., 2021a). The clinical features of TSM are complex and difficult to detect. It is often misdiagnosed and has a high mortality. After invading the human body, *T. marneffei* can be captured by macrophages to achieve the transformation from yeast phase to pathogenic phase, causing clinical diseases (Tsang et al., 2018; Hu et al., 2020; Narayanasamy et al., 2021; Pruksaphon et al., 2022). The incidence of TSM is insidious, and it can occur in all age groups. The clinical phenotype is atypical and it is easy to be misdiagnosed. The clinical phenotypes mainly include high fever, emaciation, aplastic anemia, skin lesions, hepatosplenomegaly, and diarrhea. Skin lesions are more common in patients with AIDS, mostly in the face and upper part of the trunk. The most common is central necrotic papules, which can also be manifested as nodules, cysts and ulcers (Cao et al., 2019). The treatment of TSM is mainly to kill *T. marneffei*. The main antifungal drugs are amphotericin B, itraconazole, fluconazole, and echinocandin. This article describes two cases of TSM and a literature review to provide a reference for clinical diagnosis and treatment of TSM.

## Case presentation

### Case 1

Case 1 was a 48-year-old man who was born in Jiayu County, Hubei Province, and lived in Guangzhou Province. The patient developed a fever (up to 39.5°C) in April 2019. The local hospital gave anti-infective treatment (details unspecified) but had a poor response. Thereafter, the patient developed recurrent fever and received intermittent anti-infective therapy (details unspecified) for 2 months. At the end of May 2019, the patient developed melena after taking Chinese herbal medicine and had epigastric pain, anorexia, acid regurgitation, eructation, retching, and hypodynamia. Two days later, he was admitted to the Department of Gastroenterology of our hospital. The patient had a history of herpes zoster virus infection and chronic bronchitis, was allergic to penicillin, had a 20-year history of smoking and drinking, and denied other medical histories.

**Figure 1:**
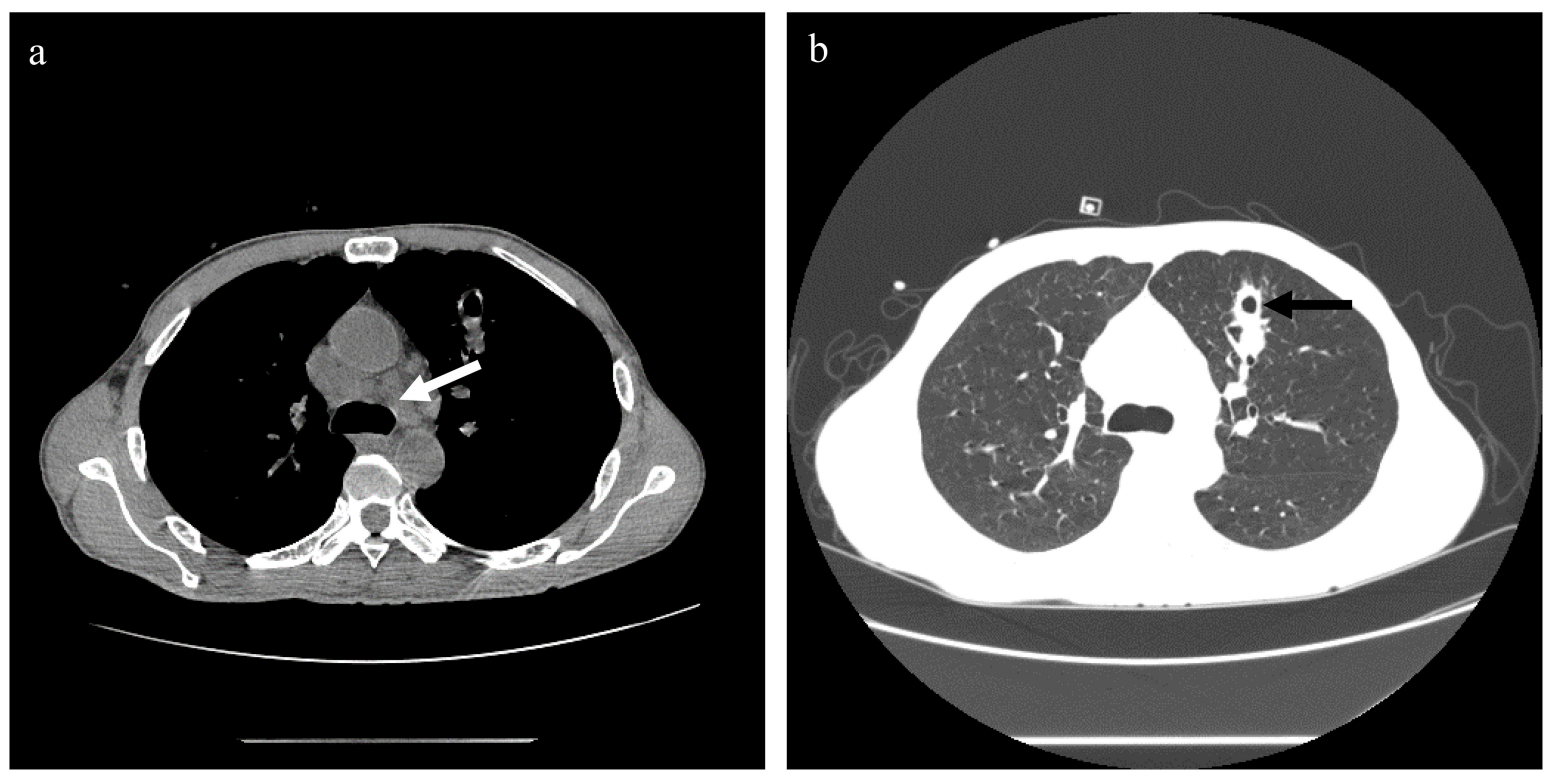
Chest CT scan of case 1. **(A)** The mediastinal window, showing multiple mediastinal lymph node enlargements (white arrow). **(B)** The lung window, showing uniform ground-glass exudation in both lungs. The left upper lung sees the irregular edge of the nodule (black arrow), and there is a cavity in the middle of the nodule. No obvious pleural effusion was found.

On admission, the patient’s blood pressure was 100/69 mmHg, body temperature was 36.4°C, pulse was 124 times/min, and respiratory rate was 17 times/min. Physical examination showed systemic skin and scleral jaundice, and slight tenderness under the xiphoid. Chest computed tomography (CT) scan showed bilateral uniform ground-glass exudation, nodules in the anterior segment of the left upper lobe, small nodules in the right lung, mediastinal lymphadenopathy, and hydropericardium (**Figure 1**).

Laboratory tests after admission showed that the patient had a systemic infection, anemia (mean red blood cell volume of 63 fL and mean red blood cell hemoglobin of 21 fL), significant reduction of platelets, hypocalcemia, liver dysfunction, biliary obstruction, coagulation dysfunction, and gastrointestinal bleeding (**Table 1**). In addition, the patient had severe cellular immune dysfunction with positive HIV-Ag/Ab. After re-asking the patient, he admitted a history of AIDS.

**Table 1:** Clinical laboratory results of case 1.

Measure	Patient on admission	Transferred to intensive care unit	Reference range
White blood cell count (10^9^/L)	6.23	4.63	3.5–9.5
Neutrophil (%)	92	85.6	40–75
Red blood cell (10^12^/L)	4.39	4.06	4.3–5.8
Hemoglobin (g/dL)	92	81	130–175
Red blood cell specific volume	0.272	0.249	0.4–0.5
Platelet count (10^9^/L)	24	34	125–350
C-reactive protein (mg/L)	>200	NA	0–10
Serumamyloid A (mg/L)	>200	NA	<10
Procalcitonin (ng/mL)	11.29	8.17	<0.05
Alanine aminotransferase (U/L)	103	137	9–50
Aspertate aminotransferase (U/L)	384	1138	15–40
Alkaline phosphatase (U/L)	331.8	NA	45–125
γ-Glutamy transpeptidase (U/L)	315	NA	10–60
Total bile acid (μmol/L)	82.85	NA	0.5–10
Total protein (g/L)	49.9	NA	65–85
Albumin (g/L)	21.3	21.51	40–55
Total bilirubin (μmol/L)	87.93	142.22	0–23
Direct bilirubin (μmol/L)	70.8	122.1	0–8
Urea (mmol/L)	NA	10.41	3.1–8
Creatinine (μmol/L)	59	NA	57–97
Ca (mmol/L)	1.7	NA	2.11–2.52
Lactate dehydrogenase (U/L)	942	NA	100–240
Prothrombin time activity (%)	59.7	28.6	75–135
APTT (s)	45.4	73.6	25–31.3
Thrombin time (s)	NA	94.9	14–21
Fibrinogen (g/L)	0.68	0.6	2–4
D-Dimer (mg/L)	72.62	95.13	0–0.5
Ammonia (μmol/L)	NA	62.8	11–32
NT-pro BNP (pg/mL)	149	3082	0–125
CD4 cell count (/µL)	27	NA	404–1,612
CD8 cell count (/µL)	64	NA	220–1,129
CD4/CD8	0.42	NA	0.9–2.0
Fecal occult blood (chem)	3+	NA	Negative
Urinary nitrite	positive	NA	Negative

**Figure 2:**
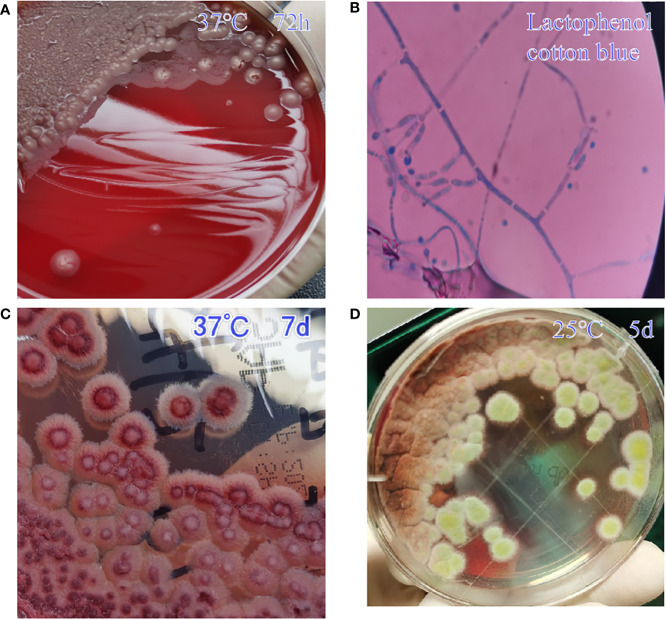
The culture performance of *T. marneffei* in case 1. **A** denotes a yeast-like colony of *T. marneffei* ( medium: blood agar plate ) grown at 37 °C for 72 hours, showing a light red villous colony with red wine on the back. **B** is the morphology of *T. marneffei* under the staining of lactophenol cotton blue. It can be seen that there are single-stranded conidia that are easy to fall off at the top of the double-wheeled broom branches (Magnification,× 1000). **C** is the state of *T. marneffei* cultured in Sabouraud Agar Plate at 37 °C for 7 days. It can be seen that milky wax-like colonies. **D** is the state of *T. marneffei* cultured in Sabouraud Agar Plate at 25 °C for 5 days. White velvet-like colonies visible.

After admission, the patient was given anti-microbial drug (cefoperazone sodium and tazobactam sodium, 4 g, q12h), antipyretic, liver protection (glutathione), acid suppression (omeprazole), hemostasis (somatostatin), and other treatments, and antiretroviral therapy (ART) was started. However, the patient still had an intermittent high fever and had general weakness, nausea, chills, sweating, and hiccups. He was given antispasmodic and cooling treatment, and the antibacterial drug was adjusted to levofloxacin. On day 4, the patient’s condition worsened; still had a fever (38.3°C); and appeared disturbance of consciousness, urine incontinence, convulsions, and inability to control limb movement. Laboratory tests showed that galactomannan (GM) test was positive; (1,3)-β-D-glucan test (G test) was 80.10 pg/mL; and urine culture, Widal test, T-SPOT.TB, tuberculosis gene, and respiratory pathogen 9 antibodies were negative. Fungal hyphae were found in the blood culture (**Figure 2**), and fluconazole was added (400 mg on the first day, 200 mg/day later). Subsequently confirmed to be T. marneffei. At this time, the patient’s liver function and coagulation function were worse, anemia was aggravated, systemic infection was still serious, and cardiac insufficiency occurred. Therefore, he was transferred to the intensive care unit (ICU) for treatment on the same day. The patient’s blood pressure was 122/86 mmHg, pulse rate was 127 beats/min, respiratory rate was 23 beats/min, and body temperature was 36.7°C at the time of admission to the ICU. Physical examination showed mental confusion and discontinuous convulsion, external genital ulcers and exudation, bilateral axillary lymph node enlargement, jaundice aggravation, liver palms of both hands, and grade 3 muscle strength of limbs. He was given coagulation factor supplementation, oxygen inhalation, supportive treatment, and adjusted the anti-infective regimen (meropenem of 500 mg q8h + micafungin sodium of 100 mg qd + voriconazole of 200 mg q12h). Re-examination of abdominal B-ultrasound showed hepatosplenomegaly, chest X-ray suggested infection of the right lower lung, and no obvious abnormality was found in the head CT scan. Blood culture, sputum culture, and bronchoalveolar lavage fluid etiology examination all returned *T. marneffei* infection. On day 5, the patient’s family asked to be discharged because of a poor prognosis, and the patient died during follow-up.

### Case 2

Case 2 was a 49-year-old man from Fuzhou, Fujian Province. The patient developed a dry cough in April 2019 and developed a fever (up to 37.7°C) 3 days later, accompanied by post-activity asthma. After 2 days of fever, he was admitted to the Department of Organ Transplantation of our hospital. The patient had a history of hypertension and uremia, long-term peritoneal dialysis, and received renal transplantation in January 2019. The patient had a history of favism, was allergic to penicillin, and denied other medical histories.

**Figure 3:**
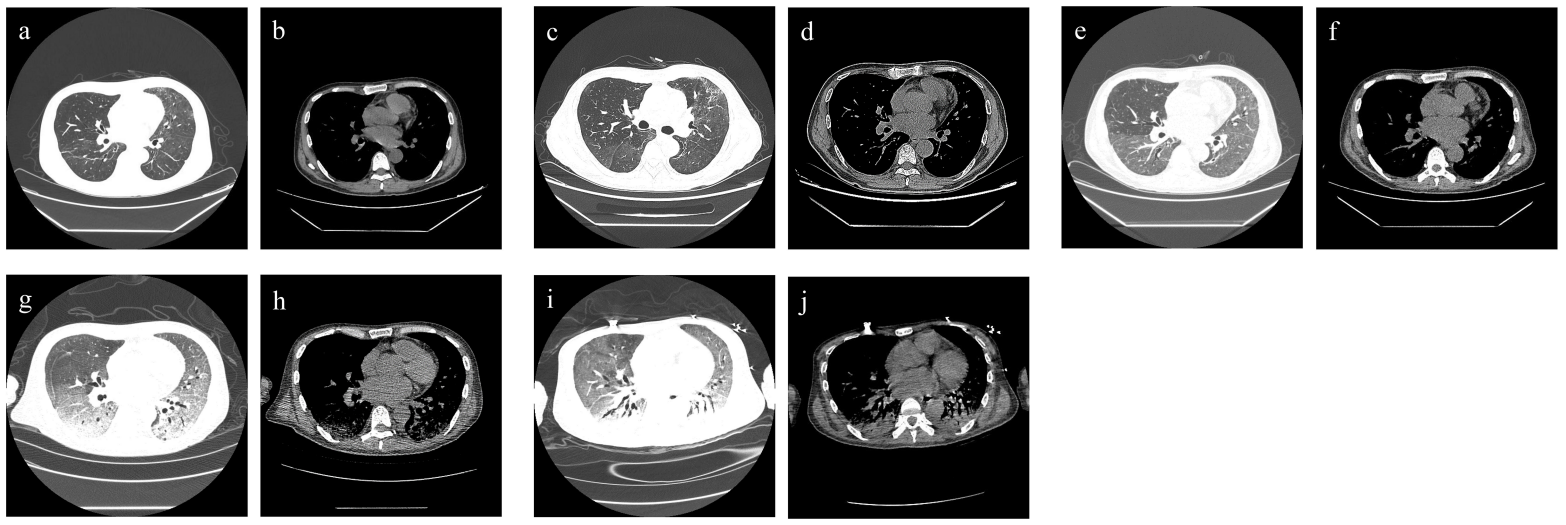
Chest CT findings of case 2. **(A, B)** For admission performance: multiple cords and patchy shadows in both lungs. **(C, D)** For the third-day performance: double lung (see multiple cords), patchy shadow, part of the range is larger than before, and the density is higher than before. **(E, F)** For the 10th-day performance: bilateral lung texture enhancement, bilateral lung parenchyma diffuse mist shadow, bilateral lung (see multiple flocculents), and cord-like density increased shadow. **(G, H)** For the 21st-day performance: bilateral lungs (see multiple large patchy density increased shadow) and fuzzy boundary. **(I, J)** For the 36th-day performance: decreased brightness of both lungs, bilateral lungs (see multiple large patchy increased density shadow), and blurred boundaries (see air bronchogram).

On admission, the patient’s blood pressure was 108/71 mmHg, respiratory rate was 20 times/min, pulse was 100 times/min, and body temperature was 37°C. Physical examination showed an abdominal renal transplantation scar, no tenderness and rebound pain, the transplanted kidney had no tenderness. Chest CT scan suggested bilateral lung infection, paraseptal emphysema of the upper lobe of both lungs, and a small amount of pericardial effusion (**Figures 3A, B**).

Laboratory tests after admission suggested that the patient had a deep fungal infection (G test of 325 pg/mL), anemia, severe thrombocytopenia, hypoproteinemia, liver and kidney dysfunction, immunodeficiency (CD4 cells, 156/µL; and CD8 cells, 64/µL), and human cytomegalovirus infection (HCMV-DNA positive) (**Table 2**). The biochemical examination results indicate a glucose-6-phosphate dehydrogenase level of 702.2 U/L. After admission, immunosuppressants (tacrolimus + mycophenolate mofetil) were discontinued, and anti-microbial drug (cefoperazone sodium and tazobactam sodium of 4 g q12h + micafungin sodium of 100 mg qd + ganciclovir of 75 mg qd), immunoglobulin infusion, gastric protection, red blood cell transfusion, cooling, and nutritional support were given. Two days later, a chest CT scan showed aggravation of bilateral lung infection (**Figures 3C, D**). A few days later, the patient developed severe asthma and fever (39°C). Respiratory pathogen detection (bronchoalveolar lavage fluid PCR) suggested *Pneumocystis jiroveci*. Therefore, the anti-infective regimen (caspofungin of 50 mg qd + imipenem cilastatin sodium of 500 mg q6h + moxifloxacin of 400 mg qd) was changed, and methylprednisolone sodium succinate (40 mg, once a day) and immunoenhancement therapy (thymopentin injection) were given. From day 5 to day 12, the patient’s vital signs were stable without fever. During the period, the chest CT scan showed extensive exudative changes in both lungs and local infectious lesions (**Figures 3E, F**). On day 13, the patient developed a fever (38°C) again, and his asthma worsened. Clindamycin phosphate ester (1 g, q12h) was added, and the dose of methylprednisolone sodium succinate was increased (40 mg, twice daily). However, the patient developed persistent fever and dyspnea and was transferred to ICU on day 15.

**Table 2:** Clinical laboratory results of case 2.

Measure	Patient on admission	Transferred to ICU	Day 18	Day 21	Day 33	Day 34	Day 35	Day 36	Reference range
Blood Pressure (mmHg)	108/71	127/97	119/80	119/80	113/81	117/84	131/80	125/90	90–140/60–90
Pulse (beats/min)	100	70	117	90	120	88	107	110	60–100
Pulse oxygen saturation (%)	NA	99	100	99	97	100	95	94	≥95
Respiratory rate (breaths/min)	20	17	22	26	35	13	29	33	12–20
White blood cell count (10^9^/L)	4.06	6.7	6.7	9.23	5.55	3.42	2.01	5.06	4.3–5.8
Neutrophil (%)	87	95.3	95.7	99.3	96.3	95.1	86.1	87.7	40–75
Red blood cell (10^12^/L)	2.55	3.09	3.13	3.08	2.89	2.63	3.26	2.55	3.5–9.5
Hemoglobin (g/dL)	72	85	87	84	86	79	96	77	130–175
Red blood cell specific volume	NA	0.269	0.271	0.277	0.265	0.236	0.283	0.222	0.4–0.5
Platelet count (10^9^/L)	24	NA	105	86	36	52	23	24	125–350
C-reactive protein (mg/L)	4.67	32.03	21.52	23.9	2.58	1.81	1.28	1.41	0–10
Serumamyloid A (mg/L)	NA	>200	>200	>200	33.1	18.97	6.22	109	<10
Procalcitonin (ng/mL)	1.28	3.06	2.1	0.625	NA	1.28	0.798	0.617	<0.05
Alanine aminotransferase (U/L)	87	22	31	38	14	27	NA	NA	9–50
Aspertate Aminotransferase (U/L)	45	37	50	55	31	66	NA	NA	15–40
Albumin (g/L)	29.7	32.82	31.4	29.9	27.6	27.9	NA	NA	40–55
Total bilirubin (μmol/L)	10.5	12.38	11.64	13.66	9.73	8.4	NA	NA	0–23
Direct bilirubin (μmol/L)	NA	5	4.2	5.2	4.6	3.8	NA	NA	0–8
Sodium (mmol/L)	NA	146	150	141	151	151	NA	NA	137–147
Potassium (mmol/L)	NA	3.7	3.96	5.95	6.42	4.42	NA	NA	3.5–5.3
Chloride (mmol/L)	NA	110.9	118.6	113.9	115.1	112.2	NA	NA	99–110
Calcium (mmol/L)	NA	2	1.95	1.97	2.18	2.22	NA	NA	2.11–2.52
Magnesium (mmol/L)	NA	1.35	1.3	1.17	1.26	1.03	NA	NA	0.75–1.02
Urea (mmol/L)	NA	38.28	40.99	25.42	56.77	35.75	NA	NA	3.1–8
Creatinine (μmol/L)	213	239	234	199	235	145	NA	NA	57–97
eGFR (mL/min)	NA	26.44	27.12	32.99	26.98	48.37	NA	NA	≥90
Prothrombin time (s)	NA	15.2	15.2	15.9	12.8	12	11.6	NA	9–13
Prothrombin time activity (%)	NA	52.7	52.7	47	66.4	73.4	81.1	NA	75–135
APTT (s)	NA	33.3	32.5	31.7	34	46.6	50.6	NA	25–31.3
Thrombin time (s)	NA	18.3	21.6	19.6	68.4	Non-clotting	138	NA	14–21
Fibrinogen (g/L)	NA	1.18	0.81	1.39	1.12	1.14	0.71	NA	2–4
D-Dimer (mg/L)	NA	13.52	15.3	9.87	3.53	3.52	3.76	NA	0–0.5
NT-pro BNP (pg/mL)	NA	3164	7773	3535	NA	2492	2011	4769	0–125
HCMV-DNA	2.63E+5	8.43E+5	NA	NA	NA	NA	NA	NA	NA
G6PD (U/L)	702.2	1870.9	1045.7	NA	1334.5	NA	NA	NA	NA

TSM, Talaromycosis; AIDS, acquired immune deficiency syndrome; CT, computed tomography; HIV, human immunodeficiency virus; APTT, activated coagulation time of whole blood; NT-pro BNP, N-terminal pro B-type natriuretic peptide; NA, not obtained; ART, antiretroviral therapy; GM test, galactomannan test; G test, (1, 3)-β-D-glucan test; ICU, intensive care unit; HCMV-DNA, human cytomegalovirus; eGFR, estimated glomerular filtration rate.

Laboratory examination at the time of admission to ICU suggested that the patient’s liver function improved, but the infection aggravated (G test of 1,493 pg/mL), anemia still existed, and coagulation dysfunction and cardiac insufficiency occurred (**Table 2**). In ICU, the patient was given non-invasive ventilator-assisted breathing, continued anti-microbial drug (cefoperazone sodium and tazobactam sodium of 4 g q12h + clindamycin of 1 g q12h + caspofungin of 50 mg qd + sulfamethoxazole of 1 g q12h + ganciclovir of 75 mg qd), diuresis, hypoglycemic, and other treatments. On day 17, imipenem (500 mg, q6h) was added. From day 17 to day 20, the patient’s vital signs were stable, but purpura appeared in the left forearm and gradually subsided after the improvement of coagulation function. On day 21, the patient developed a fever again (39°C), and a chest CT scan showed extensive exudative changes in both lungs with a bilateral lung infection, which was significantly more advanced than before (**Figures 3G, H**). Respiratory pathogen detection (bronchoalveolar lavage fluid PCR) showed *Candida parapsilosis*, the G test was 520.3 pg/mL, and GM test was positive. On day 24, the patient developed a fever again (38.1°C), and the patient’s pulmonary infection was aggravated. Therefore, the dosage of sulfamethoxazole (2 g, three times a day) and caspofungin (70 mg, once a day) was increased, and human gamma globulin was added to enhance immunity. Clindamycin and ganciclovir were discontinued. One day later, urine culture returned *Candida parapsilosis* (50,000 CFU/mL); considering the patient’s history of solid organ transplantation, prolonged use of antimicrobial agents, the presence of multiple indwelling catheters, and the failure of adequate antibacterial therapy, we point out that the patient had candidiasis. Four days later, blood culture returned *T. marneffei*. From day 29 to day 35, the patient’s vital signs were stable, with occasional afternoon low fever, and the infection was better than before, but hyperkalemia, high urea nitrogen, diarrhea, and purpura were aggravated, and the coagulation function deteriorated (**Table 2**). Renal replacement therapy and multiple transfusions of fresh frozen plasma, fibrinogen, platelets, and leukocyte-poor red blood cells were given. On day 36, the patient suddenly became irritable and could not tolerate a non-invasive ventilator. Tracheal intubation–assisted ventilation was performed, and a chest CT scan showed extensive exudative changes in both lungs, which progressed (**Figures 3I, J**). On day 37, the patient suffered sudden cardiac arrest and died.

## Discussion and conclusions

*T. marneffei* is a fungus of intracellular infection. After conidia enter the human body, *T. marneffei* can bind to the extracellular matrix through glyceraldehyde-3-phosphate dehydrogenase and adhere to the host alveolar epithelium (Tsang et al., 2018; Pruksaphon et al., 2022). Conidia can also adhere to the host extracellular matrix, fibronectin, laminin, and glycosaminoglycan through N-acetylneuraminic acid–dependent processes (Pruksaphon et al., 2022). *T. marneffei* is a temperature bidirectional fungus, which provides protection from phagocyte destruction according to morphological changes caused by temperature. Thermal dimorphism is considered to be an important virulence factor for infection (Pruksaphon et al., 2022). Although it has been found that *T. marneffei* expresses different genes and proteins in two forms, it is not clear how this transition occurs, and the *aba A* gene and Msg A protein may play an important role (Tsang et al., 2018; Pruksaphon et al., 2022). *T. marneffei* does not produce exotoxins, alveolar macrophages can recognize and phagocytize *T. marneffei* through CD11b (Hu et al., 2020). Subsequently, *T. marneffei* can replicate in macrophages in a yeast state and cause infection. In macrophages, *T. marneffei* prevents itself from being hydrolyzed by lysosomes by producing superoxide dismutase, superoxide dismutase and catalase-peroxidase and also produces potent enzymes to scavenge oxygen free radicals (Narayanasamy et al., 2021; Pruksaphon et al., 2022). It can also further evade host defense by downregulating host interleukin-6 and can capture arachidonic acid through GM protein Mp1p to destroy the host’s pro-inflammatory cascade (Narayanasamy et al., 2021). In addition, *T. marneffei* may induce the polarization of host macrophages to M2-like by inducing tyrosine phosphorylation, thereby reducing the host’s immune level (Wei et al., 2021b). In addition, melanin, mitorubrinol and mitorubrinic acid, aspartic proteases, heat shock proteins, laccases, induction of glyoxylic acid cycle at host body temperature, utilization of non-preferred nitrogen sources in the host environment, detoxification of propionyl coenzyme A using methyl citrate cycle, and isolation of host proinflammatory lipids also affect the virulence of *T. marneffei* after invading the human body (Tsang et al., 2018; Pruksaphon et al., 2022). In humans, *T. marneffei* infection can occur after acute infection and can also be latent and then activated to cause delayed infection. *T. marneffei* can invade multiple organ systems through the reticuloendothelial system, especially blood, bone marrow, and lung (Vanittanakom et al., 2006). *T. marneffei* antigen can stimulate T lymphocytes to produce cytokines such as interleukin-12, interferon-γ, and other activated macrophages (Tse et al., 2015), and CD4+ T cells are also key mediators in the anti–*T. marneffei* response (Tsang et al., 2018; Pruksaphon et al., 2022), so immunocompromised patients have a higher risk of *T. marneffei* infection. In this paper, case 1 was an HIV-infected patient, case 2 was a patient with glucose-6-phosphate dehydrogenase deficiency after renal transplantation. Both patients had immunodeficiency and were susceptible to *T. marneffei*. *T. marneffei* infection can occur at any time from 9 months to 11 years (mean of 3.68 years) after transplantation (Gupta et al., 2022). Therefore, it is necessary to consider the presence of *T. marneffei* infection when fever occurs in transplant patients.

TSM has a high incidence in Southeast Asia, northeastern India, and southern China (Vanittanakom et al., 2006). In China, 42.8% of TSM occurs in Guangxi Province and 40.6% in Guangdong Province (Hu et al., 2013). In this paper, case 1 was from Hubei but lived in Guangzhou, and case 2 was from Fujian. Both of them had a long-term residence history in the epidemic area. The clinical manifestations of patients with TSM are not typical, including fever, cough, anemia, skin lesions, hepatosplenomegaly and lymphadenopathy (Cao et al., 2019), and occasional neurological manifestations (Vanittanakom et al., 2006). Two patients in this article have different degrees of fever, fatigue, anemia, and skin lesions. Case 1 had gastrointestinal bleeding, hepatosplenomegaly, lymph node enlargement, and disturbance of consciousness. However, the patient had liver dysfunction and elevated blood ammonia, so the disturbance of consciousness may also be caused by hepatic encephalopathy. Case 2 had respiratory symptoms, dry cough, and dyspnea but had no obvious abdominal pain, diarrhea, hematochezia, and other symptoms. The chest CT findings of patients with *T. marneffei* infection were mostly cotton-like, nodular, massive, miliary, and patchy changes. Cavities could also occur, involving both lungs and mediastinal lymph node enlargement and pleural effusion. However, the above chest CT findings are not unique to TSM, so radiological changes can only be used as a diagnostic reference, not as a diagnostic criterion. HIV-positive patients with TSM are prone to cause invasive *T. marneffei* infection, affecting the function of multiple organ systems, and the mortality rate is 12%~21% (WHO fungal priority pathogens list to guide research, development and public health action, n.d). However, the mortality of HIV-negative patients with TSM is higher than that of HIV-positive patients, which may be related to delayed diagnosis and misdiagnosis (Zheng et al., 2015). In case 1, it was more than 2 months from the onset of fever to the diagnosis of TSM, and, in case 2, it was 31 days from the onset of symptoms to the diagnosis. Both patients failed to diagnose TSM in time and caused disseminated *T. marneffei* infection. In the later stage, despite active treatment, it still could not prevent the progression of the disease and eventually died. Therefore, in areas where TSM is prevalent, clinicians should screen HIV-positive patients for *T. marneffei* infection. For HIV-negative patients with immune dysfunction, clinicians should be familiar with the diagnosis and management of *T. marneffei* infection.

Both amphotericin B and voriconazole have good therapeutic effects on *T. marneffei* (Yousukh et al., 2004; Mo et al., 2014). In addition to voriconazole, triazole drugs such as posaconazole and itraconazole also have high killing activity against *T. marneffei* (except fluconazole). Amphotericin B has moderate anti–*T. marneffei* activity (Cao et al., 2019). Case 1 was initially treated with fluconazole. Although it was adjusted to voriconazole in time, the patient had a long medical history, and severe infection was difficult to control. The therapeutic effect of amphotericin B alone is better than that of itraconazole, but the incidence of adverse events such as infusion-related reactions, renal failure, and hypokalemia is also higher (Le et al., 2017). Therefore, amphotericin B combined with itraconazole is currently the most used and effective treatment (He et al., 2021). However, in less developed regions, amphotericin B is difficult to obtain and expensive, which limits its application. The medication of renal transplant patients is more difficult. Amphotericin B has nephrotoxicity, and triazole drugs interact with tacrolimus (Gupta et al., 2022). At present, there is no uniform medication standard. In case 2, the use of first-line drugs was limited, so the subsequent use of sulfonamides and caspofungin antifungal therapy may be one of the reasons for poor infection control. For HIV-positive patients, it is not clear when to start ART. Qin et al. showed that the effect of the timing of ART on prognosis was related to the use of antibiotics (Qin et al., 2022). Early ART improved prognosis in patients using amphotericin B but had no significant effect on prognosis in patients using voriconazole. In the use of antibiotics, the current recommended treatment is as follows: mild disease, itraconazole of 200 mg, oral, once every 12 hours; after 8 weeks, itraconazole of 200 mg, orally, once a day, until the CD4 cell count > 100 cells/mm3, continued ≥6 months of ART; acute infection in critically ill patients, intravenous injection of amphotericin B of 3–5 mg/kg, once every 24 hours for ≥2 weeks; then itraconazole of 200 mg oral, once every 12 hours, for ≥10 weeks; itraconazole is then orally administered at 200 mg once every 24 hours until CD4 cell count >100 cells/mm3, lasting ≥6 months, using ART (Chastain et al., 2017).

Many clinical laboratories lack experience in identifying this dimorphic fungus, and early diagnosis of TSM is difficult. *T. marneffei* shows different cell morphology at different temperatures (Lau et al., 2013), so the clear identification of *T. marneffei* needs to prove the transformation of mycelium to yeast. At present, the main methods for diagnosing *T. marneffei* infection are smear, culture, and histopathological examination, but traditional methods such as culture are time-consuming and easy to delay diagnosis, resulting in early death of patients (Zheng et al., 2015). GM test has a certain predictive value for *T. marneffei* infection (Cao et al., 2019), especially for HIV-positive patients (Zheng et al., 2015) and also has a certain predictive value for prognosis (long-term high GM test indicates poor prognosis) (Wei et al., 2021a), but the sensitivity of G test is not high (Guo et al., 2019). Studie have found that the GM optical density of HIV-infected patients with *T. marneffei* fungemia is higher than that of HIV-infected patients without fungemia and fungemia caused by other fungi (Huang et al., 2007). Therefore, the GM test may contribute to the early diagnosis of *T. marneffei* in epidemic areas. The diagnostic efficacy of non-HIV–infected patients needs further study. Serum sphingomyelin content may be helpful for the diagnosis of *T. marneffei* infection in HIV-negative patients (Li et al., 2021). Serum sphingomyelin content in patients with *T. marneffei* infection is significantly lower than that in patients with other infections. In addition, matrix-assisted laser desorption ionization time-of-flight mass spectrometry (Lau et al., 2016), inhibition enzyme-linked immunosorbent assay (Prakit et al., 2016), and other detection techniques can quickly detect *T. marneffei*. In addition, there is an immunochromatographic strip test based on asolid phase sandwich format immunoassay has been developed (Pruksaphon et al., 2021). It can directly use patient urine to detect *T. marneffei* with high specificity, sensitivity, and accuracy, but it requires a minimum antigen concentration in urine of about 0.6 μg/mL. The sensitivity in the urine of patients with TSM with negative blood culture is low, and it cannot be detected by blood samples at present. There may be some restrictions on use. Even if the current methods have their own shortcomings, but these methods for clinicians early diagnosis and treatment of *T. marneffei* infection is of great help.

TSM is a deep invasive fungal disease with high mortality caused by *T. marneffei*. Patients with immunodeficiency are the main susceptible population. Although *T. marneffei* mainly exists in South Asia and Southeast Asia, patients with TSM also occur in non-endemic areas due to frequent global exchanges. Because of the lack of awareness of the disease and because *T. marneffei* infection is difficult to accurately identify, non-epidemic area doctors often cannot give timely and effective treatment. Therefore, clinicians need to raise awareness of the disease in order to reduce the mortality of patients with TSM.

We reported two cases of TSM found in Wuhan, both of which had a history of sojourn in the epidemic area (case 1 is from Guangdong Province and case 2 is from Fujian Province). Both patients had a history of immunodeficiency. Case 1 was an HIV-positive patient, and case 2 had renal transplantation and G-6-PD, both of which were susceptible to *T. marneffei*. Both patients were disseminated patients with TSM with fever, anemia, fatigue, and skin changes and had their own unique symptoms such as gastrointestinal bleeding, hepatosplenomegaly, and psychiatric symptoms. Laboratory tests have thrombocytopenia, increased infection indicators, and coagulation dysfunction, and CD4+ cell reduction is an important feature. Chest CT showed pulmonary infection. After active treatment, all died of poor efficacy. The cases reported in this paper may help to deepen the understanding of TSM in non-endemic areas of China, as well as the understanding of treatment methods and death outcomes caused by delayed diagnosis. Improving the understanding of TSM and timely and accurate diagnosis are effective ways to reduce the mortality caused by *T. marneffei*. In the case of long culture time, PCR, gene sequencing, GM test, and antigen test paper may be the key measures for early diagnosis. We hope that this report can contribute to the realization of WHO “Fungal Priority Pathogens List.”

## Data availability statement

The original contributions presented in the study are included in the article/Supplementary Material. Further inquiries can be directed to the corresponding author.

## Ethics statement

The studies involving humans were approved by Ethics Committee of Renmin Hospital of Wuhan University. The studies were conducted in accordance with the local legislation and institutional requirements. We only collected data from the hospital ‘s electronic medical record system. Written informed consent for participation was not required from the participants or the participants’ legal guardians/next of kin in accordance with the national legislation and institutional requirements. Written informed consent was obtained from the individual(s) for the publication of any potentially identifiable images or data included in this article. Since both patients have died, the clinical details and clinical images of the two patients were released with the written informed consent of their respective close relatives.

## Author contributions

ZYY: Data curation, Formal analysis, Methodology, Software, Writing – original draft, Writing – review & editing. ZP: Data curation, Software, Writing – original draft. GL: Methodology, Supervision, Writing – review & editing. ZML: Data curation, Formal analysis, Writing – review & editing. ZY: Investigation, Software, Supervision, Writing – review & editing. LYZ: Resources, Software, Supervision, Visualization, Writing – review & editing. WFX: Investigation, Supervision, Visualization, Writing – review & editing.
